# Women’s Dental Association

**Published:** 1892-09

**Authors:** 


### WOMEN'S DENTAL ASSOCIATION.

An announcement on another page that the women dentists have perfected an organization marks an era in dentistry. It is not many years—less than a quarter of a century—since the first woman graduated from a dental college, and now they have increased sufficiently to form an organization of their own.

That this is the proper course no one, probably, will question. It has been a disappointment to some of the warmest friends of women in dentistry, that the scientific side of the profession has been so little aided by her advent into it. This has been, possibly, due to the natural retiring disposition of the sex when brought into associations composed of men, but more, doubtless, to the difficulty of securing a professional foothold in a new field. Be the cause what it may, the work of woman has not been noticeable.

We believe that this organization is a move in the right direction, and will prove an active stimulant to greater exertion in a work for which we regard woman as being especially qualified, that of scientific research.

The women dentists have now an opportunity to disarm criticism and justify the efforts of those who aided them in their earlier struggles.

